# Construction and characterization of an improved DNA-launched infectious clone of duck hepatitis a virus type 1

**DOI:** 10.1186/s12985-017-0883-5

**Published:** 2017-11-03

**Authors:** Junhao Chen, Ruihua Zhang, Shaoli Lin, Pengfei Li, Jingjing Lan, Zhijing Xie, Yu Wang, Shijin Jiang

**Affiliations:** 1 Department of Preventive Veterinary Medicine, College of Veterinary Medicine, Shandong Agricultural University, Taian, Shandong 271018 China; 2 Shandong Provincial Key Laboratory of Animal Biotechnology and Disease Control and Prevention, Taian, Shandong 271018 China; 3 Department of Basic Medical Sciences, Taishan Medical College, Shandong, Taian, 271000 China

**Keywords:** DHAV-1, DNA-launched infectious clone, Ribozyme, Rescue efficiency

## Abstract

**Background:**

DNA-launched infectious system is a useful tool with high rescue efficiency that allows the introduction of mutations in specific positions to investigate the function of an individual viral element. Rescued virus particles could be harvested by directly transfecting the DNA-launched recombinant plasmid to the host cells, which will reduce labor and experimental cost by skipping the in vitro transcription assay.

**Methods:**

A total of four overlapping fragments covering the entire viral genome were amplified and then were assembled into a transformation vector based on pIRES2-EGFP to establish the DNA-launched infectious system of duck hepatitis A virus type 1 (DHAV-1), named pIR-DHAV-1. Reverse transcription polymerase chain reaction (RT-PCR) detection, quantitative real-time polymerase chain reaction (qRT-PCR), western blotting assay and indirect immunofluorescence (IFA) were conducted for rescued virus identification. A total of 4.0 μg of recombinant plasmid of pIR-DHAV-1 and in vitro transcribed product of 4.0 μg of RNA-launched infectious clone named pR-DHAV-1 were transfected into BHK-21 cells to analyze the rescue efficiency. Following that, tissue tropism of rescued virus (rDHAV-1) and parental virus (pDHAV-1) were assayed for virulence testing in 1-day-old ducklings.

**Results:**

Rescued virus particles carry the designed genetic marker which could be harvested by directly transfecting pIR-DHAV-1 to BHK-21 cells. The qRT-PCR and western blotting results indicated that rDHAV-1 shared similar growth characteristics with pDHAV-1. Furthermore, DNA-launched infectious system possessed much higher rescue efficiency assay compared to RNA-launched infectious system. The mutation at position 3042 from T to C has no impact on viral replication and tissue tropism. From 1 h post infection (hpi) to 48 hpi, the viral RNA copies of rDHAV-1 in liver were the highest among the six tested tissues (with an exception of thymus at 6 hpi), while the viral RNA copy numbers in heart and kidney were alternately the lowest.

**Conclusion:**

We have constructed a genetically stable and highly pathogenic DNA-launched infectious clone, from which the rescued virus could be harvested by direct transfection with recombinant plasmids. rDHAV-1 shared similar growth characteristics and tissue tropism with pDHAV-1. The DNA-launched infectious system of DHAV-1 possessed higher rescue efficiency compared to the traditional RNA-launched infectious system.

## Background

Duck virus hepatitis (DVH), which was first described in Long Island in 1949 [1], **is** commonly recognized as an acute and fatal disease of ducklings. DVH is caused by duck hepatitis virus (DHV) types 1, 2 and 3, and no antigenic relationships have been found among them [2–5]. In the three different serotypes of DHVs, DHV-1 was considered as the most common and most virulent serotype [1, 6]. According to the Virus Taxonomy the Ninth Report of the International Committee on Taxonomy of Viruses (ICTV), DHV-1 was classified as a member of *Picornaviridae* and renamed as duck hepatitis A virus (DHAV) [7]. As the only member of the genus *Avihepatovirus* in the family *Picornaviridae*, DHAV has been further classified into three serotypes: the DHAV serotype 1 (DHAV-1, the classical serotype) [8, 9], the DHAV serotype 2 (DHAV-2, a serotype isolated in Taiwan) [10], and the DHAV serotype 3 (DHAV-3, a serotype isolated in South Korea and China) [11, 12]. There is no cross-neutralization between DHAV-1 and DHAV-2 [10] yet limited cross-neutralization between DHAV-1 and DHAV-3 [13]. Both DHV-2 and DHV-3 were later identified as astroviruses based on sequence analysis of a 391 nt RNA-dependent RNA polymerase [14].

DHAV-1 is a non-enveloped, single-stranded and positive-sense RNA virus, and possesses a short genome of approximately 7.7 kb excluding the poly (A) tail at the 3′ end, with a long open reading frame (ORF) flanked by 5′ and 3′ untranslated regions (UTRs) [8]. The ORF encodes a large polyprotein, which is subsequently cleaved into three capsid proteins (VP0, VP1 and VP3) and nine nonstructural proteins (2A1, 2A2, 2A3, 2B, 2C, 3A, 3B, 3C and 3D) [8]. The 5′ UTR of DHAV-1 contains an internal ribosome entry site (IRES) [15]. The 3′ UTR of DHAV-1, composed of three double-stranded hairpin stems, is 314 nt in length and is the longest among all *Picornaviridae* members [16].

Reverse genetics systems have been considered as powerful tools for studying all aspects of virus biology, including virus pathogenesis and vaccine development [17]. Up to date, there are at least four types of viral RNA rescued system, including RNA-launched system [18], helper virus-launched system [19] and two DNA-launched systems based on cellular RNA polymerases I and II [20–22]. Although the infectious DHAV genomic RNA could be harvested in vitro from a full-length DHAV cDNA clone by the RNA-launched reverse genetics system [23, 24], the operative strategy still depends on in vitro transcription. Compared to the RNA-launched infectious clone, the RNA polymerases II-based DNA-launched infectious system is able to generate homogenous RNA transcripts from transfected cDNA clone in vivo*,* allowing for higher rescue efficiency with less cost and labor by skipping in vitro RNA transcription [25–27]. For porcine reproductive and respiratory syndrome virus (PRRSV), by the introduction of ribozyme elements at both termini of the viral genomic cDNA, an improved DNA-launched infectious clone improved the rescue efficacy with approximately 10–50-fold higher than the in vitro-transcribed RNA-based system [28]. The hammerhead ribozyme is a catalytic motif responsible for the self-cleaving activity of RNA virusoids [29], while the hepatitis delta virus ribozyme also contains site-specific self-cleavage with the existence of divalent cations and a cytidine nucleotide [30]. After transcription, the ribozymes at both termini of DNA-launched infectious clones could lead into the self-cutting and releasing of the viral mRNA under the condition of existing Mg^2+^ or other divalent ions and a cytidine nucleotide. By this way, the viral RNA can be harvested by direct transfection with the recombinant plasmid without in vitro transcription [31].

In the previous study, a highly pneumovirulent strain of DHAV-1, LY0801 (originally named LY01), was isolated from a 7-day-old duckling exhibiting typical duck viral hepatitis (DVH) in 2008 from an outbreak of severe DVH in Shandong province, China [9]. Based on the virulent strain LY0801, we firstly established a DNA-launched infectious clone of DHAV-1, which possessed higher rescue efficiency compared to the traditional RNA-launched infectious system.

## Methods

### Cell, viruses and antibodies

BHK-21 cells were cultured at 37 °C in 5% CO_2_ in minimum essential medium (MEM) supplemented with 10% fetal bovine serum (FBS), 100 units/ml penicillin, and 100 μg/ml Streptomycin Sulfate. The construction of this infectious cDNA clone of DHAV-1 was based on the 5th passage of LY0801 strain (accession no. FJ436047). LY0801 is a virulent strain of DHAV-1 isolated in 2008 from an outbreak of severe DVH in Shandong province, China [9]. The LY0801 virus produced an acute and fatal disease in one-week-old ducklings. The mortality of duck flock was 80%, and the sick ducklings died quickly with typical hemorrhagic hepatitis. After propagated in the allantonic cavities of 10-day-old duck embryos and adapted to BHK-21 cells for five passages, the LY0801 strain was used for the full-length genomic cDNA construction. Anti-DHAV-1 monoclonal antibody (mAb) 4F8, which could recognize the epitope “_75_GEIILT_80_” in VP1 of DHAV-1, was stored in the veterinary molecular etiology laboratory of Shandong Agricultural University [32]. Fluorescein isothiocyanate (FITC)-labeled goat anti-mouse antibody was purchased from KPL (MD, USA), Horseradish Peroxidase (HRP)-labeled goat anti-mouse antibody was purchased from Abcam (MA, USA).

### Cloning and sequencing the full-length genome of DHAV-1

To determine the complete sequence of LY0801 strain, the viral RNA was extracted from allantoic liquids of dead duck embryos with a E.Z.N.A.™ Viral RNA Kit (Omega Bio-Tek, Norcross, GA, USA) followed by RNA transcription using RevertAid™ First Strand cDNA Synthesis Kit (Fermentas, Burlington, Canada) according to the manufacturer′s instruction. Nine pairs of specific primers were designed to amplify 9 overlapping PCR fragments (Table 1). PCR products were purified using a gel purification kit (Invitrogen, CA, USA), and then were TA-cloned into the pMD18-T vector (TaKaRa, Dalian, China) following the manufacturer′s protocol. The TA-cloned products were then transfected into *E. coli* DH5α competent cells (TaKaRa, Dalian, China), and the positive clones were selected by PCR amplification and then were sent to a commercial service for sequencing with dideoxy terminal termination method by 3730xl DNA Analyzer (ThermoFisher, MA, USA) by Sangon Biotech Co., Ltd., Shanghai, China. The 5′ sequences of the viral genome were determined by 5′ rapid amplification of cDNA ends (RACE). The complete genomic sequence of the 5th passage of the DHAV-1 LY0801 strain in BHK-21 cell culture was compared with the FJ436047 sequence by the Clustal W method (DNA Star LaserGene software, DNAStar Inc. Madison, WI) and then used for the construction of DNA-launched infectious clone.

**Table 1:** PCR primers used in this study

Primer	Primer sequences (5′ → 3′)
DHAV-Seq-1F	TTT GAA AGC GGG TGC AT
DHAV-Seq-1R	TTA TAG TGT GTG GGA CTC GAC C
DHAV-Seq-2F	GAC TAG TTC CTG AGG GAC AGA TGT T
DHAV-Seq-2R	TCC CTG ATT GTC AAA TGG TCG G
DHAV-Seq-3F	ACA ACT GGT GGT GCC ATT TGT GT
DHAV-Seq-3R	CTG CCA AAA GTT GCC TCT GAT GTG C
DHAV-Seq-4F	TGG ATG ACC TCA CTT CAG AGT ATG C
DHAV-Seq-4R	TTG ACT GCA TGT GAT CAC CTG CTG G
DHAV-Seq-5F	TAA ATG GTG AAG TCA CAA TCA AG
DHAV-Seq-5R	GGC CAA AAT CAT CAA AAG CAT
DHAV-Seq-6F	CTC TTG GTA TAT GGA TAT CTG GTG GT
DHAV-Seq-6R	GTA GCA ATC AAT TTA GAC ACA TCT
DHAV-Seq-7F	AGA TGA GAT TAG GGA CAT
DHAV-Seq-7R	TGG GTA TAA CAT CAC TAC TC
DHAV-Seq-8F	AGA CAC ATG TTG CTG AAA AAC T
DHAV-Seq-8R	AGA ACA CAG TCA TCC CCA TAA CT
DHAV-Seq-9F	CTG ATG AGA TAT GGC AGG TA
DHAV-Seq-9R	TTT TTT TTT AGG TAG GGT AGG GAA T
pIR-BamHI-XhoI-F	GGG ATC CTA CTC TAG ACA TAA TCA GCC ATA CCA CAT TTG TAG AGG
pIR-BamHI-AscI-R	GAA GGA TCC ACT GGC GCG CCT CGA GAT CTG AGT CCG GTA G
5HeadRibo-F	ACA TGG CGC GCC *ACA TCA TCT GAT GAG TCC GTG AGG ACG AAA CGG TAC CCG CGT GAG GAC GAA ACG GTA CCC GGT ACC GTC A*TT TTG AAA GCG GGT GCA TGC ATG GCC AT
5HeadRibo-R	AGG TGG ATC CAC TAT TGT CAC CTT C
3endRibo-F	CTG ATG AGA TAT GGC AGG TA
3endRibo-R	GCT CTA GA*G TCC CAT TCG CCA TTA CCG AGG GGA CGG TCC CCT CGG AAT GTT GCC CAG CCG GCG CCA GCG AGG AGG CTG GGA CCA TGC CGG CC*T TTT TTT TTT TTT TTT TTT TTA GGT AGG GTA GGG AAT AGT AAA GT
Cloning 2-F	TAG TGG ATC CAC CTG AAA CAC C
Cloning 2-R	ACC AGAT ATC CAT ATA CCA AGA GGT T
BamH I-detect-F	ACA ACT GGT GGT GCC ATT TGT GT
BamH I-detect-R	TTG ACT GCA TGT GAT CAC CTG CTG G
RT-qPCR-F	AGA CAC ATG TTG CTG AAA AAC T
RT-qPCR-R	AGA ACC AGT TGT CGT TTG GTC
DHAV-1-Probe	Cy5-ATG CCA TGA CAC TAT CTC ATA TGA GTC AGC-BHQ-2

### Assembly of the full-length cDNA clone

**Fig. 1:**
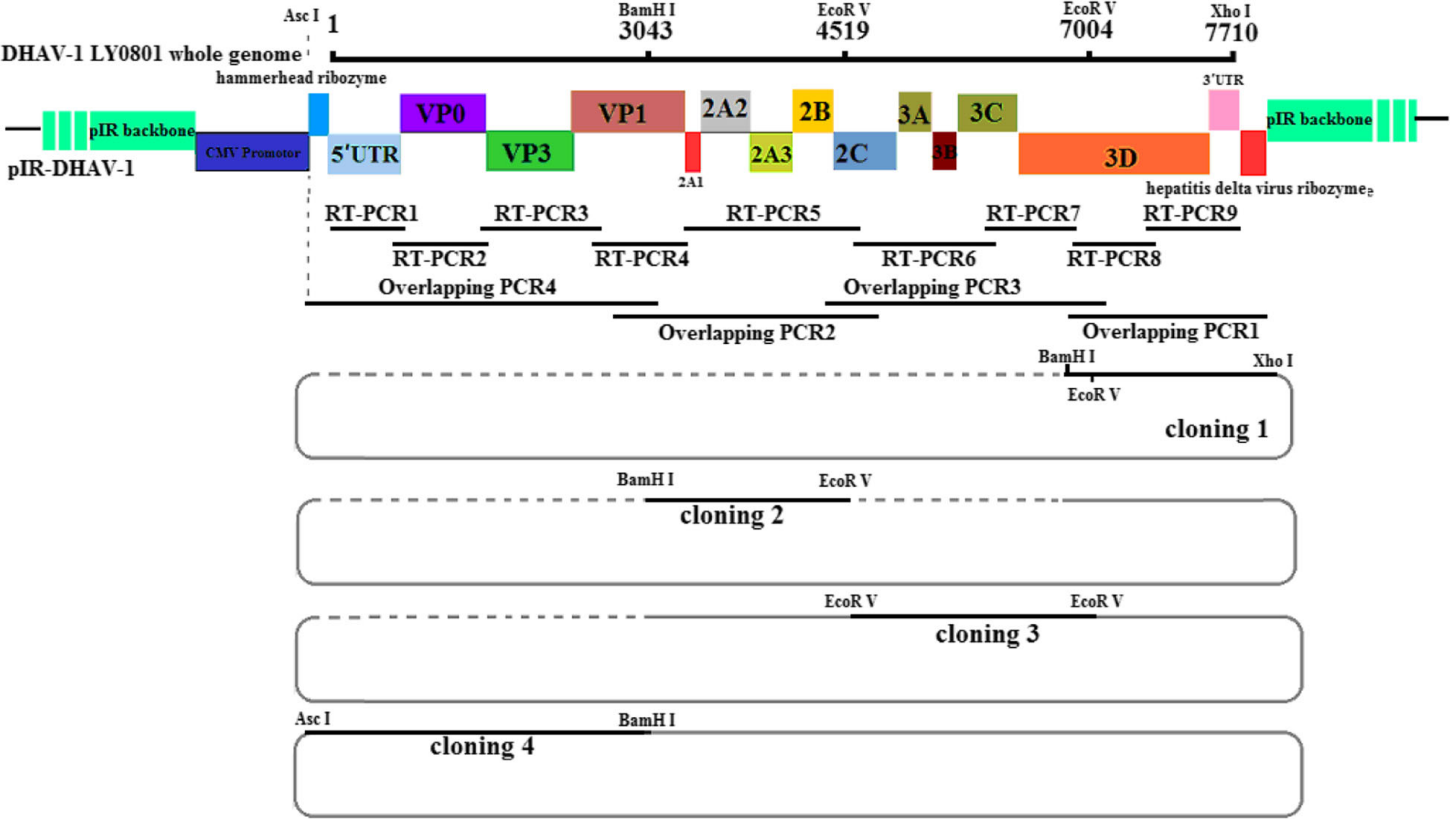
Strategy for the construction of a full-length DNA-launched infectious clone based on a virulent strain LY0801 of DHAV-1. The fragment IR was derived from the plasmid pIRES2-EGFP (Clontech) using primers pIR-BamHI-XhoIF and pIR-AscI-BamHIR, and then was digested with *Bam*H I and ligated with T4 DNA ligase to yield pIR vector. The LY0801 virus genome with two ribozyme sequences introduced at the both ends was placed downstream of the CMV promoter in pIR vector to construct the DNA-launched infectious clone, pIR-DHAV-1. A total of 4 overlapping fragments amplified from total RNAs extracted from the 5th passage of LY0801 virus were assembled successively into the pIR vector by unique restriction enzyme indicated for each fragment. A copy of hammerhead ribozyme sequence and Asc I restrict enzyme site were engineered at the 5′ end while a copy of hepatitis delta virus ribozyme (HDVRs) sequence and *Xho* I restrict enzyme site were engineered at the 3′ end of the cDNA copy of DHAV full-length genome, respectively. The base T at position of 3042 was mutated into base C to generate a *Bam*H I restrict enzyme site which was treated as a genetic marker to distinguish the rescued virus from the parental virus

A total of 9 overlapping fragments covering the entire viral genome based on the cDNA of the 5th passage of LY0801 strain were amplified for construction of DNA-launched infectious clone. The fragment IR was amplified with the primers pIR-BamHI-XhoIF/pIR-BamHI-AscI-R (Table 1) based on the vector pIRES2-EGFP (Clontech, Mountain View, CA) to introduce corresponding enzyme restriction sites at both terminuses. The fragments were then digested with *Bam*H I and ligated with T4 DNA ligase (TaKaRa, Dalian, China), yielding a vector named pIR to delete enhanced green fluorescent protein (EGFP) gene. The fragments clone 1 containing the hammerhead ribozyme and 1 to 3042 of LY0801 sequence was amplified with 5HeadRibo-F/R, while the clone 4 possessing the hepatitis delta virus ribozyme and 6820 to 7710 of viral sequence was amplified with 3HeadRibo-F/R, respectively (Fig. 1). The ribozyme sequences were shown in italic in Table 1. By overlapping PCR, four fragments covering the full-length genome were amplified with the four pairs of primers, namely 5HeadRibo-F/R (clone 1), Cloning 2-F/R (clone 2), DHAV-Seq-6F/9R (clone 3) and 3endRibo-F/R (clone 4) (Table 1). The four fragments were assembled into pIR vector by one multi-step strategy (Fig. 1).

The fragment cloning 1 and pIR vector was digested with *Bam*H I and *Xho* I and then ligated by T4 DNA ligase. The ligation products were then transformed into *E. coli* DH5α competent cells, yielding a recombinant plasmid named pIR-1. By introducing an *Eco*R V restriction enzyme site into pIR-1 through ligation of cloning 1, a newly recombinant plasmid named pIR-12 was obtained by transforming the ligation of *Bam*H I/ *Eco*R V*-*digested products of pIR-1 and fragment cloning 2 into *E. coli* DH5α competent cells. The fragment cloning 3 was amplified with primer DHAV-Seq-6F/9R, including two *Eco*R V restriction enzyme sites on both terminuses (lie in position 4517–4522 and 7002–7007 of viral genome). After digestion of *Eco*R V*,* the fragment cloning 3 was inserted into vector pIR-12, yielding the recombinant plasmid pIR-123. The base at the position 3042 was mutated from T to C (the mutation causing no amino acid sequence change) by the designed primers to generate a *Bam*H I restrict enzyme site, which was used as a genetic marker to distinguish the rescued virus from the parental virus. Following that, fragment cloning 4 was inserted into pIR-123 after digestion with *Asc*I and *Bam*H I. The DNA-launched infectious clone of DHAV-1 was named as pIR-DHAV-1.

### Transfection of BHK-21 cells

When reaching around 70% confluence in a six-well plate, BHK-21 cells were transfected with 4.0 μg of purified plasmid pIR-DHAV-1 per well using 10 μl Lipofectamine 2000 Transfection Reagent (Invitrogen) according to the manufacturer′s protocols. After incubation for 4 h, the cells were washed by phosphate-buffered saline (PBS; 8.1 mM Na_2_HPO_4_, 1.5 mM KH_2_PO_4_, 140 mM NaC1, 3.0 mM KC1, pH 7.2) for five times and fresh MEM with 2% FBS was added to each well.

### Detection of the rescued virus by IFA

At 60 h post transfection (hpt), the rescued viruses were harvested and propagated in BHK-21 cells. Equal amount of pDHAV-1 was propagated in BHK-21 cells as positive control while the same amount of PBS was conducted as the negative control. At 48 hpi, the cells were fixed by a mixture of acetone and formaldehyde (1:1) for 30 min at room temperature. After washed five times with PBS, the cells were incubated with anti-DHAV-1 mAb 4F8 (dilution of 1:1000 with PBS) for 1 h at 37 °C. FITC-labeled goat anti-mouse antibody (dilution of 1:1000 with PBS) was used as secondary-antibody and the cells were incubated at 37 °C for 1 h in the dark. The stained cells were analyzed by fluorescence microscopy (Leica AF6000).

### SDS-PAGE and western blotting

To further confirm whether the infectious virus particles could be harvested from DNA-launched infectious system, the transfected product was used to propagate in BHK-21 cells. The supernatants were collected and centrifuged at 3000 rpm for 5 min at 4 °C to remove the floating cells and debris. The BHK-21 cells were manually harvested by a cell scraper, washed for three times with PBS and resuspended with equal amount of PBS. For Western blot analysis of cell culture media, cells were collected as previously described, washed three times with PBS and then resuspended with supernatant. Cell lysates (200 μl) were then added with 50 μl 5× SDS loading buffer and boiled at 100 °C for 10 min and incubated for 5 min on ice. The samples were run on SDS 12%-polyacrylamide gels and transferred onto a polyvinylidene fluoride (PVDF) membrane (ThermoFisher, MA, USA) by standard procedures. Following that, the PVDF membrane was blocked in 5% non-fat milk in Tris-Buffered Saline saline Tween-20 (TBST) (500 ml NaCl, 0.05% Tween 20, 10 mM TRIS-HCl pH 7.5) for 1 h on a horizontal table. Anti-DHAV-1 monoclonal antibody 4F8 (dilution of 1:500) was incubated at 4 °C for 8 h. The membrane was washed four times with TBST and incubated with HRP-conjugated goat anti-mouse antibody (Abcam, MA, USA, dilution of 1:3000) at 4 °C for 4 h. The PVDF membrane was then visualized with hydrogen peroxide and 3, 3′-diaminobenzidine tetrahydrochloride (DAB) (Sigma, USA). The BHK-21 cells infected with pDHAV-1 or with pIR vector were conducted as positive and negative controls, respectively.

### Identification of the genetic marker in rescued virus

During the construction of the recombinant plasmid, the base T at position 3042 was replaced by base C to create a genetic marker to distinguish rDHAV-1 from the parental virus pDHAV-1. The transfected products were harvested at 60 hpt and were immediately used for RNA extraction as previously described. Following that, total RNA was used for RT-PCR amplification with primers *Bam*H I-detect-F/R (Table 1) diluted in RNAase free water (TaKaRa, Dalian, China). RT-PCR/PCR was performed utilizing a one-step RNA PCR kit (TaKaRa, Dalian, China). The RT-PCR/PCR condition was 50 °C for 30 min, 94 °C for 2 min, and then 30 cycles of 94 °C for 30 s, 50 °C for 30 s, 72 °C for 1 min, and with a final step of 72 °C for 5 min. During the RNA extraction assay, the DNaseI Digestion kit (OMEGA, GA, USA) was used to remove the residual recombinant plasmid. The 2069 bp fragment including the genetic marker was then digested by restriction enzyme *Bam*H I and verified by electrophoresis in 2% agarose gel.

### Comparison of growth characteristics between the parental virus and the rescued virus

To examine the growth characteristics of the rescued virus, BHK-21 cells were respectively infected with pDHAV-1 and rDHAV-1 at 0.1 MOI (multiplicity of infection, with viral copies of 4.71 × 10^4^ and cell numbers of 4.69 × 10^5^), and incubated at 37 °C in 5% CO_2_. MEM supplemented with 10% FBS was added to each well 2 hpt, and the cells were cultured at 37 °C in 5% CO2. The supernatant (1.0 ml) was collected every 12 h from 12 hpi to 72 hpi, while the cells were scraped from the plate and resuspended with the same amount of PBS. The cell culture samples were collected by scraping the cells with a cell scraper and resuspended with the supernatant. The samples were treated with three freeze-thaw cycles, and the equal amounts of samples were immediately used for RNA extraction. The extracted RNA was then used for qRT-PCR amplification as previously described [33]. With the primers RT-qPCR-F/R and the DHAV-1-Probe (Table 1), a TaqMan real-time RT-PCR assay for quantitative detection of DHAV-1 was conducted in a total volume of 25 μl, containing 12.5 μl 2 × One step RT-qPCR buffer (with ROX), 0.4 μM of forward primer and reverse primer, 0.2 μM of probe, 0.9 μl EnzyMix, 2 μl template RNA. PCR was initiated by 95 °C denaturation for 5 min followed by 40 cycles at 95 °C for 15 s, annealing at 60 °C for 45 s, and fluorescence was measured at every annealing step. Non-template control (NTC) samples were included in each run. The supernatants and cells samples from pDHAV-1 and rDHAV-1 group at each detection point were also conducted with western blotting assay as previously described. A 10-mm glass coverslip was put into the 6-well plate before the equal number of BHK-21 cells were seeded in the 6-well plate. When reaching around 70% confluence, the glass coverslip was taken out from the plate and was immediately used for cell counting with Countess II FL (ThermoFisher, MA, USA).

### Comparison of rescue efficiency between RNA- and DNA-launched infectious clones

The RNA-launched infectious clone of DHAV-1 of LY0801 strain (named as pR-DHAV-1) was established previously by introducing a sp6 promoter following with a copy of DHAV-1 genome into pcDNA™3.1/V5-His A vector. The recombinant plasmids of RNA-launched infectious clone were linearized at the 3′ terminus of viral genome with *Xho* I and then were purified according to the manufacturer′s instruction (OMEGA, Norcross, GA, USA). The purified product was measured with a spectrophotometer (Eppendorf, Germany) to determine the concentration, and then used for in vitro transcription using SP6 RiboMAX™ Express LargeScale RNA Production System (Promega, USA) following the manufacturer′s instruction. RNase-free DNase I (TaKaRa, Dalian, China) was added into in vitro transcribed products and incubated at 37 °C for 15 min to digest residual DNA template, and the in vitro transcribed RNA was then purified with RNeasy kits (QIAGEN). During the construction of the RNA-launched infectious clone, the base A at position 3949 was replaced by C to generate a unique restriction site *Bam*H I to distinguish the rescued virus from parental virus. To identify the rescue efficiency of pR-DHAV-1 and pIR-DHAV-1, 4.0 μg of recombinant plasmid of pIR-DHAV-1 or the in vitro transcribed RNA of 4.0 μg of pR-DHAV-1 were transfected into BHK-21 cells. At 48 hpt, IFA assay was conducted with mAb 4F8 and FITC-labeled goat anti-mouse antibody to measure the difference on rescue efficiency. The experiment was repeated three times. The same amount of pIR empty vector was transfected into BHK-21 cells as the negative control. In order to identify the difference in rescue efficiency between the DNA-launched infectious system and RNA-launched infectious system, cell cultures of the two groups were collected at 48 hpt to conduct the western blotting assay.

To compare the rescue efficiency between the pR-DHAV-1 and pIR-DHAV-1 infectious system on infectious virus level, the cell lysates in the both transfection groups were collected and treated with three freeze-thaw cycles, and then were used to infect BHK-21 cells. Cell lysates (generation 1) were collected at 48 hpi, and 100 μl of cell lysate was used for qRT-PCR measurement while the rest 900 μl was used to infect BHK-21 cells. The rescued virus propagation characteristics of pR-DHAV-1 and pIR-DHAV-1 from generation 1 to 5 were measured by qRT-PCR.

### Duckling virulence study of rDHAV-1

Thirty 1-day-old ducklings were divided into three groups and then inoculated intramuscularly with 0.25 ml of 10^4.0^ LD_50_ of rDHAV-1, 10^4.0^ LD_50_ of pDHAV-1 of strain LY0801 and aseptic PBS (pH 7.2), respectively. The ducklings were kept under the optimal condition, clinical signs and mortality were observed daily. Ducks received analgesia (carprofen, 5 mg kg^−1^, i.p.) before the start of surgery. Based on that, ducks were anaesthetized with isoflurane (3.5%) and subjected to execution. Total RNA was extracted from the liver of the dead ducklings for RT-PCR using the primers BamH I-detect-F/R (Table 1). The amplified fragments were digested with restriction enzyme *Bam*H I and then were verified by electrophoresis in 2% agarose gel, and then were sent to a commercial service for sequencing with methods and instrument by Sangon Biotech Co., Ltd., Shanghai, China. The nucleotide sequence and amino acid sequence in both rDHAV-1 group and pDHAV-1 group were analyzed by the Clustal W method (DNA Star LaserGene software, DNAStar Inc. Madison, WI).

The animal experiments were carried out in accordance with the guidelines issued by Shandong Agricultural University Animal Care and Use Committee (SDAUA-2014-014).

### Dynamic analysis of viral load

Ducks received analgesia (carprofen, 5 mg kg^−1^, i.p.) before the start of surgery. Ducks were anaesthetized with isoflurane (3.5%) and maintained under anaesthesia (2–2.5%) throughout the inoculation. Fifty 1-day-old healthy ducklings were injected subcutaneously with 0.25 ml of 10^4.0^ LD_50_ of rDHAV-1. The heart, liver, kidney, spleen, thymus and bursa of Fabricius (BF) of the 1-day-old ducklings were collected at 1, 6, 12, 18, 24, 48 and 72 h post inoculation. According to the manufacturer′s instruction, virus RNA was isolated from tissue samples using the E.Z.N.A.™ Viral RNA Kit (Omega Bio-Tek, Doraville, USA) and then were measured by qRT-PCR. Non-template control (NTC) samples were included in each run.

The animal experiments were carried out in accordance with the guidelines issued by Shandong Agricultural University Animal Care and Use Committee (SDAUA-2014-014).

## Results and discussion

### Sequence comparison

A sequence comparison between the complete genomic sequence of the fifth passage of the DHAV-1 LY0801 strain in BHK-21 cell culture and the FJ436047 sequence was performed. Both the 3′ end poly(A) tail of the two viral strains were 21 bases in length. Comparing with FJ436047, there were six base changes after 5 passages: A changed to G at nucleotide position 1289 in *VP0* gene, C to G at nucleotide position 2397 in *VP1* gene, G to A at nucleotide position 3566 and T to C at nucleotide position 3587 and in *2A3* gene, G to A at nucleotide position 5699 in *3C* gene, and A to G at nucleotide position 6839 in *3D* gene. These mutations caused no change of the amino acids (Table 2). Comparing with the 5th passage of LY0801 strain, there was no base change in pIR-DHAV-1 or rDHAV-1 except for the mutation (from T to C) at 3042 position.

**Table 2:** Summary of sequence differences between LY0801 strain and 5th passage of LY0801 strain

Base position^a^	LY0801 strain	5th passage of LY0801	Amino acid change	Location
1289	A	G	silent	VP0
2397	C	G	silent	VP1
3566	G	A	silent	2A3
3587	T	C	silent	2A3
5699	G	A	silent	3C
6839	A	G	silent	3D

^a^Nucleotide position and sequence are based on LY0801 strain (GenBank accession no. FJ436047)

### Detection of the rescued virus

**Fig. 2:**
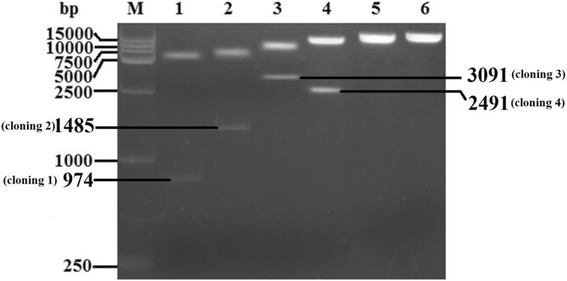
Recombinant plasmids digested with appropriate enzymes during construction. Four fragments that amplified with the four pairs primers 3endRibo-F/R, Cloning 2-F/R, DHAV-Seq-6F/9R and 5HeadRibo-F/R were assembled into pIR vector by one multi-step strategy, respectively. The yield recombinant plasmids were digested with appropriate enzymes and verified by electrophoresis in 2% agarose gel. (M) DNA Marker DL2000; (1) The recombinant plasmid that consisted of pIR vector and cloning 1 was digested with *Bam*H I and *Xho* I; (2) The recombinant plasmid that consisted of pIR vector and cloning 1 and cloning 2 was digested with BamH I and *Eco*R V; (3) The recombinant plasmid that consisted of pIR vector and cloning 1 to cloning 3 was digested with *Eco*R V; (4) The complete DNA-launched infectious clone was digested with *Asc* I and *Bam*H I; (5) The complete DNA-launched infectious clone digested with BamH I; (6) The complete DNA-launched infectious clone digested with *Xho* I

**Fig. 3:**
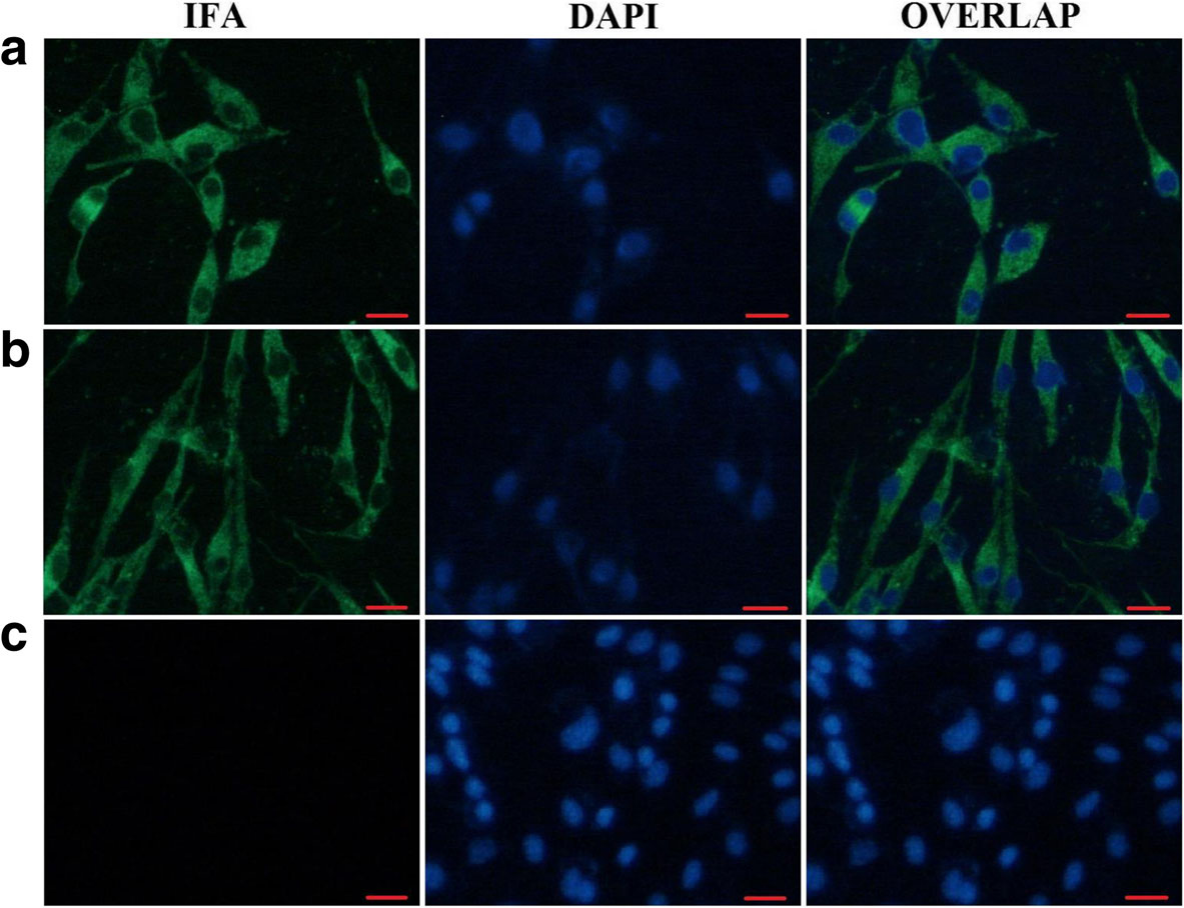
IFA observation of transfected/infected BHK-21 cells. (**a**) BHK-21 cells were transfected with the recombinant plasmid DNA of pIR-DHAV-1; (**b**) BHK-21 cells were infected by the parental virus of LY0801 strain; (**c**) BHK-21 cells were transfected with the plasmid DNA of pIR. After passaging for three times, IFA was conducted by incubation of anti-DHAV-1 mAb 4F8 (dilution of 1:1000 with PBS) for 1 h at 37 °C, followed by incubation in 37 °C for 1 h with FITC-labeled goat anti-mouse antibody. Red bars represent 10 μm

**Fig. 4:**
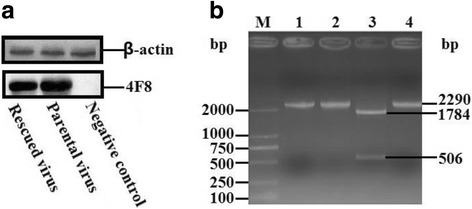
Identification of the rescued virus. (**a**) Western blotting assay. The BHK-21 cells infected with parental virus and rescued viruses were harvested at 48 hpi. Anti-DHAV-1 monoclonal antibody 4F8 (dilution of 1:500) and HRP-conjugated goat anti-mouse antibody (dilution of 1:3000) were used to conduct the western blot assay. The BHK-21 cells transfected with pIR vector were used as negative control. (**b**) Identification of the genetic marker in the rescued virus. The *Bam*H I restriction enzyme site was introduced into the recombinant plasmid to create a genetic marker to distinguish the rescued virus from the parental virus (without *Bam*H I enzyme site). Two 2290 bp-fragments derived from parental and rescued virus with primers DHAV-3F and DHAV-4R were digested with *Bam*H I and analyzed on a 2.0% agarose gel. (M) DNA Marker DL2000; 1. Fragment amplified with template of parental RNA; 2. Fragment derived from the parental virus digested with *Bam*H I; 3. Fragment derived from the rescued virus digested with *Bam*H I; 4. Fragment amplified with template of rescued virus RNA

The full-length cDNA clone of the 5th passage of LY0801 strain was assembled into the vector pIR by inserting four overlapping fragments into pIR vector using the restriction sites *Bam*H I at nucleotide position 3042, *Eco*R V at 4519 and 7004, and *Asc* I and *Xho* I at both terminuses. The recombinant plasmids were digested with appropriate enzymes and verified by electrophoresis in 2% agarose gel (Fig. 2). The monolayer BHK-21 cells that infected with the transfection product of recombinant plasmid pIR-DHAV-1 was detected by IFA with anti-DHAV-1 monoclonal antibody 4F8. The green fluorescence indicated the viral protein expression in the pIR-DHAV-1 group and positive control group, no specific green fluorescence was observed in the negative control group (Fig. 3). In order to confirm the rescued virus could be harvested from the DNA-launched infectious system, western blot assay was conducted with anti-DHAV-1 monoclonal antibody 4F8. The replication level of viral particles of pDHAV-1 and rDHAV-1 at 48 hpi was determined. Western blot result indicated that rescued virus could be rescued by direct transfection with pIR-DHAV-1 (Fig. 4A).

### Identification of the genetic marker in rescued virus

In order to distinguish rDHAV-1 from pDHAV-1, nucleotide T at position 3042 was mutated into C to create a restriction enzyme site *Bam*H I as genetic marker (Fig. 1). Fragments including the genetic marker were amplified by RT-PCR from RNA extraction products of the rescued virus and the parental virus with primers BamH I-detect-F and BamH I-detect-R (Table 1). After digested by *Bam*H I, the fragment amplified from the rDHAV-1 was cleaved into a fragment of 1784 bp and a fragment of 506 bp, while the fragment amplified from the pDHAV-1 could not be cleaved into two fragments (Fig. 4B).

### rDHAV-1 and pDHAV-1 shared similar growth characteristics

**Fig. 5:**
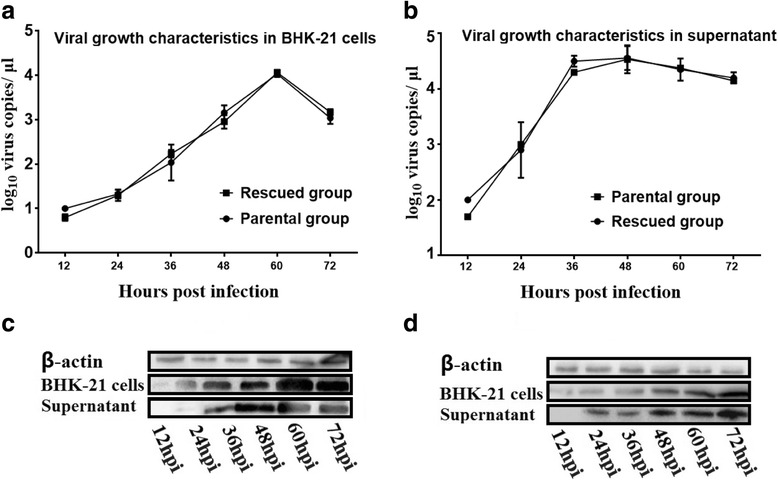
Identification the growth characteristics of rDHAV-1. BHK-21 cells were infected with the parental virus and the rescued virus at 0.1 MOI, respectively. Supernatants and cells were separately collected and used for RNA extraction and qRT-PCR amplification and western blotting. (**a**) Growth curves of the parental/rescued virus in supernatants; (**b**) Growth characteristics of the parental/rescued virus in BHK-21 cells. (**c**) Viral replication levels of the rescued viruses in supernatants and BHK-21 cells. (**d**) Viral replication levels of the parental viruses in supernatants and BHK-21 cells

To compare the growth characteristics between rDHAV-1 and pDHAV-1, the growth kinetics of the two viruses were analyzed by infection of BHK-21 cells with the respective virus at 0.1 MOI. From 12 hpi to 72 hpi, the rDHAV-1 showed similar replication and infectivity efficiency with the pDHAV-1 in supernatants (Fig. 5A) and in BHK-21 cells (Fig. 5B). The viral RNA copies peaked at 60 hpt in cells and 48 hpt in supernatants. Western blotting results showed that the viral protein expression level in BHK-21 cells was higher compared to the supernatants group, and the capsid protein levels in pDHAV-1 and rDHAV-1 group were equally similar at each monitoring point (Fig. 5C, D). These data demonstrated that the rDHAV-1 and pDHAV-1 shared similar growth characteristics in both supernatants and BHK-21 cells.

### Rescue efficiency of RNA- and DNA-launched infectious clone

**Fig. 6:**
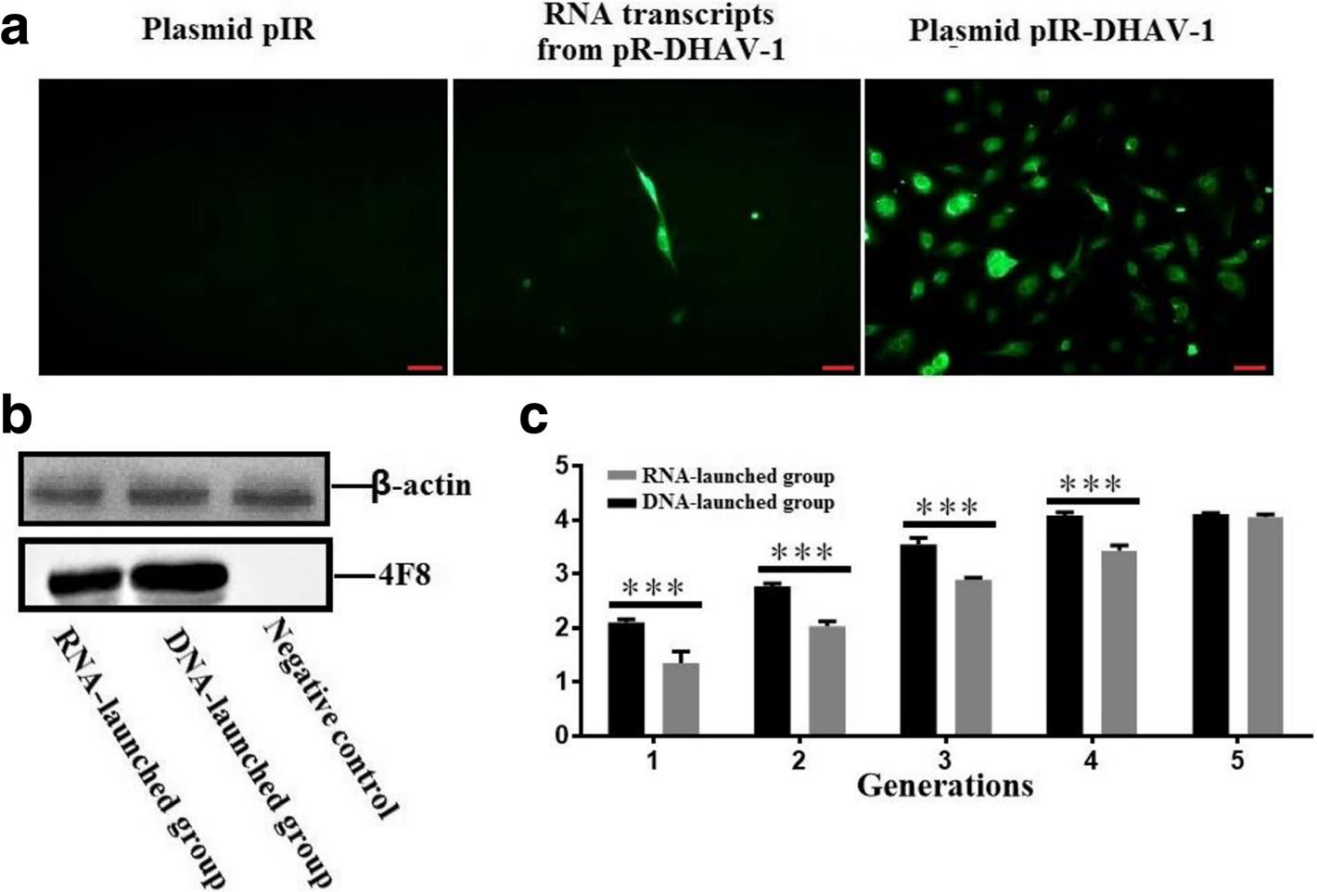
Rescue efficiency of RNA/DNA-launched infectious system. (**a**) 4.0 μg of the DNA-launched infectious clones or in vitro transcribed product of the same amount of RNA-launched infectious clones were used for BHK-21 transfection. The DNA-launched infectious clone (plasmid pIR-DHAV-1) and the empty vector (plasmid pIR) were transfected directly into BHK-21 cells, while the RNA-launched infectious clone was transcribed in vitro, and then the transcription mixture was transfected into BHK-21 cells. At 48 hpt, IFA observation was conducted after staining with anti-DHAV-1 mAb 4F8 (dilution of 1:1000 with PBS) for 1 h at 37 °C, followed by incubation in 37 °C for 1 h with FITC-labeled goat anti-mouse antibody. Bars represent 10 μm. (**b**) Western blotting assay. A total of 4.0 μg of recombinant plasmid of DNA-launched infectious clone and in vitro transcribed product from 4.0 μg of RNA-launched infectious clone were transfected into BHK-21 cells to analyze rescue efficiency between DNA-launched system and RNA-launched system. Cell culture was collected at 48 hpt and run on SDS 12%-polyacrylamide gels. Anti-DHAV-1 monoclonal antibody 4F8 (dilution of 1:500) and HRP-conjugated goat anti-mouse antibody (dilution of 1:3000) were used to conduct the western blot assay. BHK-21 cells that transfected with pIR vector were conducted as negative control. (**c**) BHK-21 cells were infected with transfected product of 4.0 μg of the DNA-launched infectious clones or in vitro transcribed product of the same amount of RNA-launched infectious clones, cell lysates were collected at 48 hpi, and were immediately used for RNA extraction and qRT-PCR measurement. Viral RNA copies were calculated with formula X = 6.7 × 10(40.812-y)/3.285. “X” is a standard of viral copies while “y” is a standard of values derived from one step real-time PCR. Bars represent means and standard of three individual repeats. “***” represents *p* < 0.001

The rescue efficiency of pR-DHAV-1 and pIR-DHAV-1 was measured by IFA method. The green fluorescence was first observed at 18 hpt in pIR-DHAV-1 group, whereas weak green fluorescence was first observed at 24 hpt in the pR-DHAV-1 group. At 48 hpt, approximately 3–5% of the BHK-21 cells transfected with the pR-DHAV-1 RNA transcripts presented specific green fluorescence, whereas a much higher percentage of FITC-positive cells, approximately 50–60%, were observed when transfected with the pIR-DHAV-1 plasmid DNA (Fig. 6A). Furthermore, we investigated expression levels of viral capsid proteins by western blotting assay. Obviously, the viral capsid protein concentration derived from pIR-DHAV-1 group was much higher than the pR-DHAV-1 group (Fig. 6B), indicating that the DNA-launched infectious system provided more sufficient viral capsid proteins for packaging infectious virions comparing to the RNA-launched system.

To compare the rescue efficiency between the RNA-launched infectious system and DNA-launched infectious system, the rescued viral particles from both groups were used to infect BHK-21 cells, and the viral growth characteristics in both groups were measured with qRT-PCR. The results showed that the virus RNA copy number in pIR-DHAV-1 group was constantly higher compared to the pR-DHAV-1 group from generation 1 to generation 4 (Fig. 6C), which demonstrated the higher rescue efficiency of the DNA-launched infectious system than the RNA-launched infectious system.

### Virulence of rDHAV-1 and pDHAV-1

**Fig. 7:**
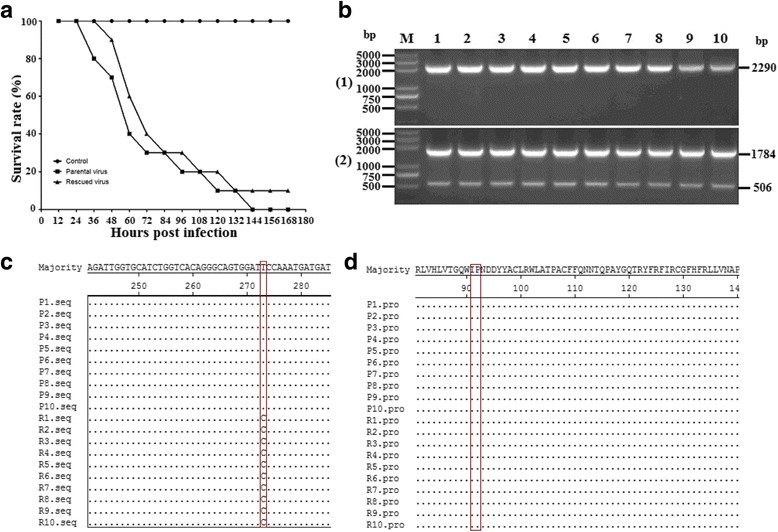
(**a**) Survival curves of 1-day ducklings after inoculation of 0.25 ml of the parental virus (10^4.0^ TCID_50_) or the rescued virus (10^4.0^ TCID_50_). All ten ducklings in control group live in healthy condition. (**b**) Genetic marker detection of the parental/rescued virus group. Total RNA was extracted from liver tissue of dead ducklings of the rescued virus group and the parental virus group, and then immediately used for transcription using the primers BamH I-detect-F/R. The amplified fragments were then digested with restriction enzyme *Bam*H I and verified by electrophoresis in 2% agarose gel. (M) DNA Marker DL2000; (1) Fragments were amplified with the template RNAs of the parental virus group and then digested with *Bam*H I; (2) Fragments were amplified with the template RNAs of the rescued virus group and then digested with *Bam*H I. Nucleotide sequence alignment (**c**) and amino acid sequence alignment (**d**) were also conducted by the Clustal W method (DNA Star LaserGene software, DNAStar Inc. Madison, WI) to investigate genetic marker in both groups

The virulence of the rDHAV-1 and pDHAV-1 were tested in 1-day-old ducklings. By identification of the specific genetic marker in rDHAV-1, the ducklings were verified to be infected with the rDHAV-1 or pDHAV-1 and there was no cross-infection with each other (Fig. 7A). A similar pattern occurred in the survival curves of the ducklings inoculated with the same dose of the two viruses (Fig. 7B). At 7 days post inoculation, all the ten ducklings died in the pDHAV-1 group, while nine of the ten ducklings died in the rDHAV-1 group. The nucleotide sequence alignment results showed that all the amplified fragments contain the mutation at the position of 3042 in the rDHAV-1 group, yet not in the pDHAV-1 group (Fig. 7C). The amino acid sequence alignment results indicated that the silent mutation has no impact on viral protein synthesis (Fig. 7D).

### Dynamic analysis of viral load

**Fig. 8:**
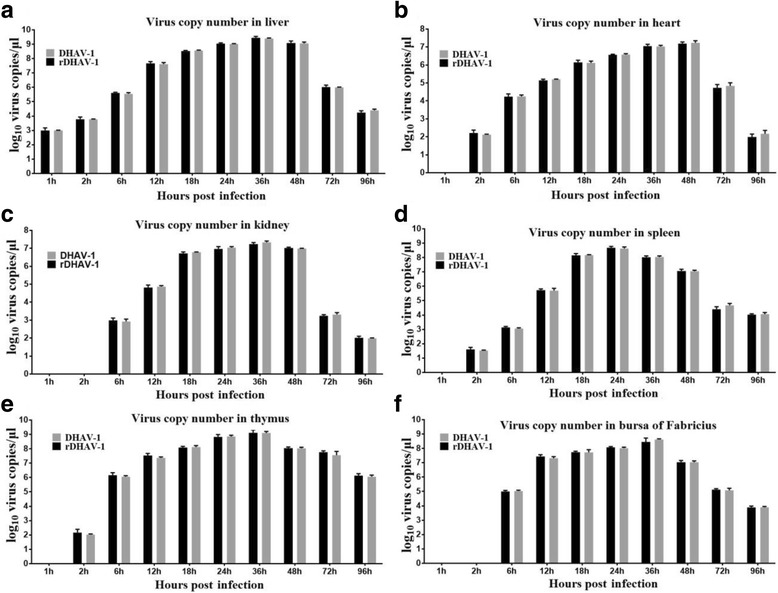
Dynamic analysis of viral load. Same weight of heart (**a**), liver (**b**), kidney (**c**), spleen (**d**), thymus (**e**) and bursa of Fabricius (**f**) of the 1-day-old ducklings were collected at 1, 6, 12, 18, 24, 48 and 72 hpi, and were immediately used for RNA extraction and qRT-PCR measurement as previously described. Viral RNA copies were calculated with formula X = 6.7 × 10(40.812-y)/3.285. “X” is a standard of viral copies while “y” is a standard of values derived from one step real-time PCR. Bars represent means and standard of three individual repeats

At 1 hpi, the viral RNA of rDHAV-1 was exclusively detected in the liver, reaching the level of 10^3.0 ± 0.0^ copies/g (Fig. 8A). The rDHAV-1 was first detected in the heart, spleen and thymus at 2 hpi, while the viral RNA was detectable from 6 hpi to 96 hpi in all the collected tissues (Fig. 8). The viral loads increased sharply and reached the peak at 36 hpi in liver, kidney, BF and thymus, whereas the viral loads in spleen and heart reached the peak at 24 hpi and 48 hpi, respectively. From 1 hpi to 48 hpi, the viral RNA copies of rDHAV-1 in liver were the highest among the six tissues (with an exception of thymus at 6 hpi), while the viral RNA copy numbers in heart and kidney were alternately the lowest. Interestingly, the detection result at 96 hpi showed that the viral RNA copies of rDHAV-1 in thymus still remain a high viral load level of 10^6.06 ± 0.1^ copies/g (Fig. 8). The similar curve pattern in the pDHAV-1 group indicated us that the genetic marker causes no impact on viral replication and tissue tropism.

## Discussion

Reverse genetics system is particularly useful for RNA viruses since RNA genomes are difficult to manipulate directly. Although the previous research has reported about the establishment of the RNA-launched infectious clone of DHAV-1 [23] and DHAV-3 [24], the in vitro transcription will raise the operation difficulty and experiment cost. The DNA-launched infectious system possessed self-cleaving ribozyme elements at both termini of the viral genomic cDNA, which was expected to retain the authentic terminal nucleotide sequences of the viral genome [28]. The 3′ product of the HDV ribozyme was shown to serve as the substrate for polyadenylation [34]. Here, we established an improved DNA-launched infectious system based on the DHAV-1 LY0801 strain. The viral genome of DHAV-1 was placed under the control of a CMV promoter, allowing the conventional plasmid DNA transfection and generation of homogenous RNA transcripts in vivo by the cellular RNA polymerase II, which could reduce labor and experimental cost. Compared to the RNA-launched infectious system, the DNA-launched infectious system of DHAV-1 improved the viral rescue efficiency approximately 10 to 20 folds (Fig. 6).

The rDHAV-1 and pDHAV-1 shared similar biological characteristics in BHK-21 cells in terms of replication efficiency, the generation of CPE (cytopathic effect), as well as infectivity (Fig. 5). Meanwhile, a similar pattern also occurred in the survival curves of the ducklings inoculated with the rDHAV-1 or pDHAV-1 (Fig. 7A). In order to distinguish the rescued virus from parental virus, the genetic marker was engineered into pIR-DHAV-1. The virus copy number peaked at 48 and 60 hpi in supernatants and BHK-21 cells, respectively. The virus copy number was subsequently followed by a decline, which was consistent with the observation of parental group. Western blot results also confirmed the similar growth characteristics between the rescued virus and parental virus (Fig. 5). The survival curve comparison between the rDHAV-1 and pDHAV-1 also showed almost identical infectivity characteristics. To sum up, the rescued recombinant virus was capable of infecting susceptible host cells and exhibited almost identical replication and infectivity efficiency as 5th passage of LY0801 strain. Based on that, we then investigated viral replication features of rDHAV-1 in young ducklings. In the previous study**,** the viral RNA could be detected in all the different tissues (heart, liver, kidney, spleen, thymus and BF) of the duckling inoculated with the rDHAV-1, which was consistent with the naturally infected ducks by DHAV-1 [33]. In our experiment, the viral RNA of rDHAV-1 was firstly detected in liver with viral load of 10^3.0 ± 0.0^ copies/g at 1 hpi, whereas the rDHAV-1 was detected in the other five tissues at 2 hpi or 6 hpi (Table 2), which showed that liver might be the most sensitive tissue during the early infection of DHAV-1 via subcutaneously. With an exception of thymus at 6 hpi, the liver tissue exhibited the highest viral load in the six selected tissues from 1hpi to 48 hpi, which could partly explain the severe liver damage induced by the infection of DHAV-1.

## Conclusions

We have described an improved DNA-launched reverse genetics system for DHAV-1 with high rescue efficiency, which can be used to save human and financial resources. The rescued virus and parental virus shared similar biological properties and pathogenicity. The DNA-launched reverse genetics system allows the rapid introduction of mutations into the viral genome, which will greatly facilitate the study of the structural and functional relationship of DHAV-1 genes, as well as the development of DHAV-1 vaccine based on the genetically engineered and attenuated mutant clones.

## Abbreviation

BHK: Baby Hamster Syrian Kidney
CPE: Cytopathic effect
DAB: Diaminobenzidine tetrahydrochloride
DHAV: Duck hepatitis A virus
DVH: Duck virus hepatitis
EGFP: Enhanced Green Fluorescent Protein
FBS: Fetal Bovine Serum
FITC: fluorescein isothiocyanate
hpi: hours post infection
hpt: hours post transfection
HRP: Horseradish Peroxidase
IFA: Indirect Immunofluorescence
LD_50_: Lethal dose 50%
MEM: Minimum essential medium
MOI: Multiplicity of infection
ORF: Open reading frame
PRRSV: Porcine reproductive and respiratory syndrome virus
PVDF: Polyvinylidene fluoride
qRT-PCR: Quantitative real time polymerase chain reaction
RACE: rapid amplification of cDNA ends
RT-PCR: reverse transcription polymerase Chain Reaction
SDS: Sodium dodecyl sulfate
TBST: Tris-Buffered Saline saline Tween-20
UTR: Untranslated regions
